# SARS-CoV-2-host and interactions: the dual roles of E3 ubiquitin ligases and ubiquitin-like modification mechanisms in viral infection

**DOI:** 10.3389/fimmu.2026.1796416

**Published:** 2026-06-12

**Authors:** Jingyi Fu, Zhizhong Mi, Zhaolong Li

**Affiliations:** 1 Institute of Virology and AIDS Research, The First Hospital of Jilin University, Changchun, Jilin, China; 2 Department of Infectious Diseases, Infectious Diseases and Pathogen Biology Center, Key Laboratory of Organ Regeneration and Transplantation of The Ministry of Education, The First Hospital of Jilin University, Changchun, Jilin, China

**Keywords:** ACE2, E3 ubiquitin ligases, SARS-CoV-2, spike protein, tmprss2

## Abstract

Following the global outbreak of the COVID-19 pandemic, the interactions between SARS-CoV-2 and host cells have attracted widespread attention. As crucial intracellular enzymes, E3 ubiquitin ligases are involved in numerous physiological processes, including protein degradation, cell cycle regulation, and immune responses. Recent studies have demonstrated that E3 ubiquitin ligases play a pivotal role in the interplay between SARS-CoV-2 and the host. Through interactions with host E3 enzymes, SARS-CoV-2 regulates key processes such as viral replication, immune evasion, and apoptosis. For instance, viral proteins can bind to E3 enzymes to modulate host immune responses and inhibit interferon production, thereby promoting persistent infection. Conversely, E3 enzymes can also regulate the viral life cycle and host cell survival by mediating targeted protein degradation. This mini-review summarizes the roles of E3 ubiquitin ligases in SARS-CoV-2 infection, introduces E3 ligase-mediated ubiquitin-like modifications, and discusses their underlying mechanisms at the virus-host interface. Furthermore, we highlight future research directions and potential therapeutic strategies. Understanding the functions of E3 ubiquitin ligases not only provides novel insights into the pathogenesis of SARS-CoV-2 but also offers promising targets for the development of antiviral therapeutics.

## 1 Introduction

SARS-CoV-2 (severe acute respiratory syndrome coronavirus 2) is the pathogen responsible for the COVID-19 pandemic, which has exerted an unprecedented impact on global health and the economy. Although vaccines and antiviral therapies have been instrumental in mitigating the pandemic, the high mutability of SARS-CoV-2 and its complex immune escape mechanisms still pose challenges to pandemic control. Most current interventions target viral proteins themselves and are susceptible to failure due to viral mutations. This underscores the strategic value of targeting conserved host pathways such as the ubiquitin system. Consequently, gaining an in-depth understanding of virus-host interactions particularly how the virus exploits host immune regulatory mechanisms to promote its own replication-has become a major research focus.

The immune response induced by SARS-CoV infection can be likened to a molecular “arms race” between the virus and the host, with E3 ubiquitin ligases acting as critical molecular switches. These enzymes are deeply integrated into the viral life cycle via precise ubiquitination modifications. For example, MARCH8 promotes the ubiquitination and degradation of the SARS-CoV-2 envelope (E) (1), while the E3 ubiquitin ligase RNF5 targets the E protein to inhibit viral replication (2). However, this system is not a unidirectional defense barrier. SARS-CoV-2 cleverly employs a “molecular mimicry” to hijack some host E3 enzymes, negatively regulating or degrading key immune signaling proteins to achieve immune evasion.

Despite these findings, most studies have focused on binary interactions between individual E3 ligases and specific viral proteins, leaving a gap in our systemic understanding of the synergistic or antagonistic networks between antiviral and proviral E3 ligases. The evolutionary strategies by which the virus balances host enzyme hijacking with evasion of host defenses remain poorly understood. Therefore, a core battleground in combating SARS-CoV-2 infection is the antagonistic interplay between “antiviral E3s” (which protect the host) and “proviral E3s” (which are recruited by the virus).

A critical challenge in current research is distinguishing targetable proviral E3 ligases from essential E3 ligases required for normal host physiology. Excessive inhibition or activation of E3 ligases can lead to immune dysregulation, such as autoinflammatory responses, posing a significant hurdle for drug specificity. It is thus imperative to elucidate the spatiotemporal specificity of these complex interaction networks, including the functional switching of E3 ligases across different stages of infection. Resolving this network will not only advance our understanding of viral pathology but also provide innovative strategies for host-directed therapies aiming to restore antiviral immune balance.

Tissue- and cell type-specific E3 ligases also influence the symptoms and severity of diseases, and may have long-term effects on patients. Differential expression of tissue- and cell type-specific E3 ligases can significantly impact the severity of COVID-19 and Long COVID. In alveolar cells, E3 ligases such as TRIM25 and RNF5 determine the efficiency of RIG-I/MAVS-mediated IFN induction; insufficient expression leads to enhanced viral replication and ARDS. In immune cells, imbalances in A20 (TNFAIP3), ITCH, and Cbl-b amplify NF-κB and inflammasome responses, promoting cytokine storms. In the nervous system, Parkin (PRKN) mediates mitophagy; reduced function may result in ROS accumulation, neuroinflammation, and brain fog. Abnormalities in endothelium-related E3 ligases such as SMURF1 and MDM2 are associated with microthrombosis and fibrosis. Moreover, SARS-CoV-2 variants further enhance host adaptation by remodeling interactions with these E3 ligases and the ISG15/SUMO systems (3–7).

In addition, E3 ligase-mediated ubiquitin-like modifications also play an important role in the game between the host and the virus. However, the crosstalk mechanisms between such modifications and ubiquitination, as well as whether viruses can simultaneously manipulate multiple ubiquitin-like modification systems to achieve immune evasion, remain largely unexplored at present. Based on the in-depth understanding of the virus and the mechanism of the struggle between the host and the virus, the development of specific antiviral drugs has become possible.

## 2 E3 ubiquitin ligases: key executors of host antiviral defense and viral immune escape

The emergence of E3 ubiquitin ligases as central regulators in SARS-CoV-2 infection is deeply rooted in their fundamental role in the ubiquitin-proteasome system. Ubiquitination is a critical post-translational modification that governs a wide spectrum of cellular processes, predominantly proteasome-mediated degradation. This ATP-dependent enzymatic cascade is driven by the sequential action of three core components: ubiquitin-activating enzymes (E1), ubiquitin-conjugating enzymes (E2), and ubiquitin ligases (E3) (8–10). Within this triad, E3 ligases act as the ultimate determinants of substrate specificity, positioning them as the core orchestrators of the intracellular ubiquitin network. Consequently, they have emerged as highly promising therapeutic targets, particularly in the rapidly advancing field of targeted protein degradation (TPD). Based on their distinct structural domains and catalytic mechanisms, E3 ligases are broadly categorized into three major families: RING (Really Interesting New Gene), HECT (Homologous to the E6-AP Carboxyl Terminus), and RBR (RING-Between-RING) (11).

The centrality of E3 ubiquitin ligases in SARS-CoV-2-host interactions stems from their modular structures and highly specific regulatory capabilities. For example, the typical RING domain directly catalyzes or recruits ubiquitination machinery to dictate the fate of substrate proteins (e.g., activation, degradation, or relocalization). In the context of antiviral immunity, this precision enables E3 ligases to act as rapid switches that initiate or amplify key pathways, such as interferon signaling. To survive, viruses inherently target these powerful host regulators, aiming to dismantle immune defenses by structurally or functionally impairing them.

However, viral evolution has progressed beyond merely disrupting E3 ligase function. SARS-CoV-2 not only hijacks E3 ligases via molecular mimicry but also exploits the functional redundancy within the E3 ligase family. If one proviral E3 ligase is inhibited, the virus can activate a functionally analogous enzyme to maintain replication. This compensatory mechanism complicates the development of E3-targeted therapeutics. Furthermore, the central role of E3 ligases makes them a double-edged sword: while their precise regulation is vital for host defense, their aberrant activation or inhibition can result in autoimmunity or immunodeficiency. Thus, the virus-host conflict fundamentally represents a battle for the regulatory balance of E3 ligases, rather than simply their binary activation or inhibition. PROTACs appear to be a feasible approach. They are bifunctional molecules that bind a viral protein at one end and recruit a host E3 ligase at the other, leading to K48-linked ubiquitination and subsequent degradation of the viral protein (12). Mpro, PLpro, N protein, and ORF9b can all serve as targets (13, 14). Compared to their widespread use in cancer therapy, the application of PROTACs in E3 ligase-mediated antiviral therapy also presents some potential issues. This is because viral proteins are more unstable than cancer-related targets, and the presence of PROTACs may also over-occupy host E3 ligases, thereby exerting adverse effects on the host. One solution could focus on virus-specific surface ternary complex structures, fully exploiting the dependency of E3 ubiquitin ligases on the correct spatial configuration of lysine residues. Additionally, tissue-restricted E3 ligases could be recruited, which would reduce systemic side effects and minimize off-target degradation of host proteins.

## 3 Interaction network at the host-virus interface

To study how E3 ubiquitin ligases mediate between promoting and inhibiting viral infection, it is necessary to first understand how the host and the virus interact.

The viral life cycle begins with the recognition, attachment, and invasion of host cells. Angiotensin-converting enzyme 2 (ACE2) is the primary cellular receptor for both SARS-CoV and SARS-CoV-2; however, SARS-CoV-2 exhibits a significantly higher binding affinity for ACE2, contributing to its enhanced infectivity. The spike (S) protein of SARS-CoV-2 binds to ACE2 via its S1 subunit, initiating viral entry either through membrane fusion or receptor-mediated endocytosis (15–17). Transmembrane protein 106B (TMEM106B), predominantly located in late endosomes and lysosomes, can act as an alternative receptor. It interacts directly with the receptor-binding domain (RBD) of the S protein, facilitating invasion even in ACE2-deficient cells (18–22).

**Figure 1:**
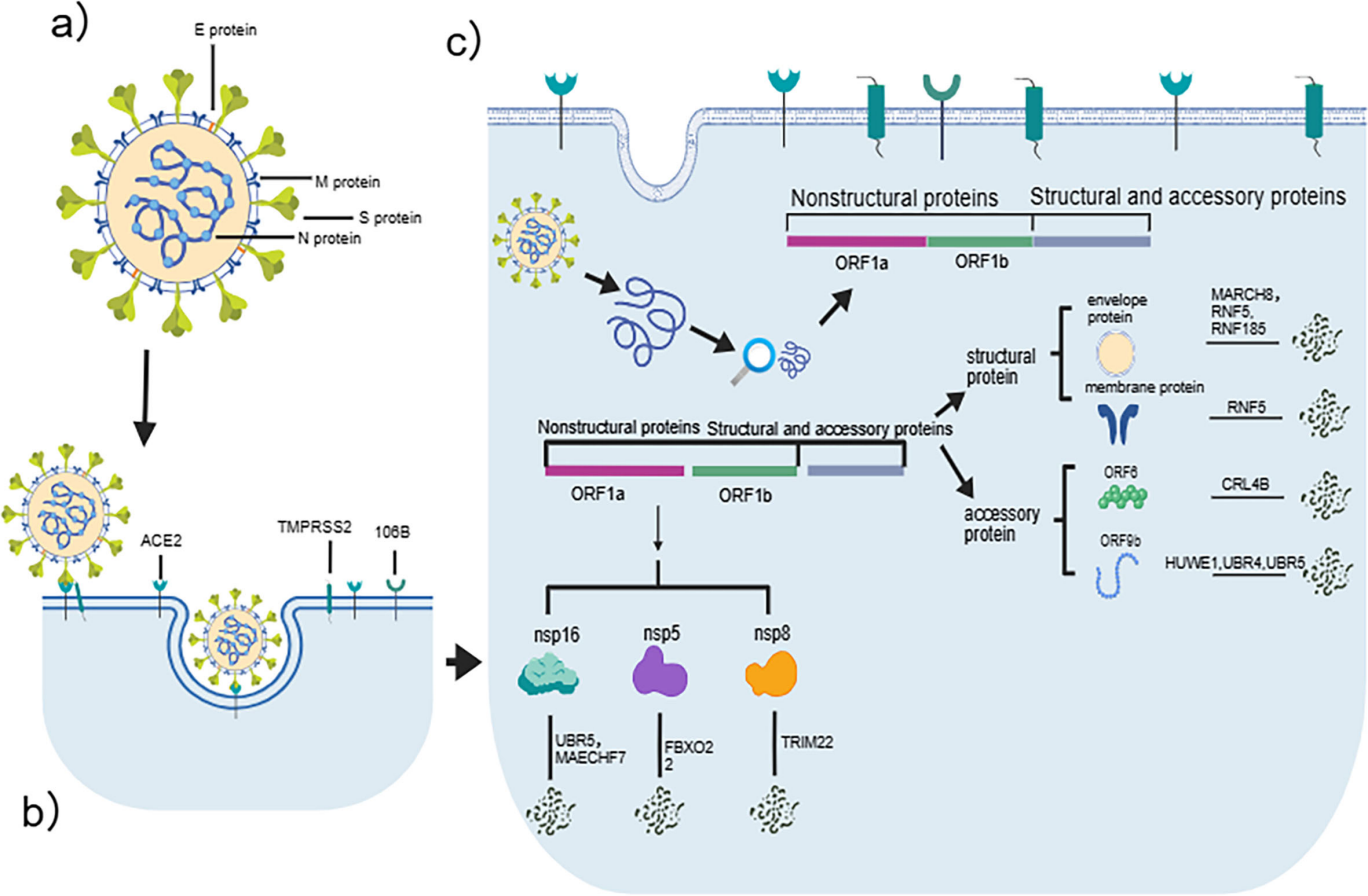
**Figure 1** covers viral entry into host cells, the structure of the viral genetic material, and the proteins targeted by various E3 ubiquitin ligases. **(A)** The viral structure shows the distribution of E, M, S, and N proteins (15–17). **(B)** Viral entry: the S protein binds to ACE2. TMPRSS2 promotes fusion of the virus with the host cell membrane by cleaving the S protein. 106B facilitates virus–cell fusion (18–21). **(C)** After entering the cell, the virus replicates and produces various proteins that perform distinct functions. Different E3 ubiquitin ligases degrade specific target proteins, as detailed in **Table 1**. (Created with BioGDP.com).

TMPRSS2, a transmembrane serine protease abundant in respiratory epithelial cells, is critical for viral entry. It cleaves the S protein, priming it for membrane fusion (23). Endogenous inhibitors like antithrombin (AT) is an inhibitor of TMPRSS2 that can be effectively activated by heparin to prevent the invasion of SARS-CoV-2 and other coronaviruses, showing its potential as an endogenous inhibitor (24). In addition, adaptor protein complex AP-1 involved in regulating clathrin-coated vesicle formation and endosomal sorting, influences viral entry. Studies have shown that certain AP-1 complex subunits can inhibit the entry of SARS-CoV-2 through the cell surface pathway, and their mechanism of action may be related to regulating the localization of the transmembrane serine protease TMPRSS2 (25, 26). Schematic diagram of SARS-CoV-2 entering cells refers to **Figure 1**.

Upon cellular entry, SARS-CoV-2 hijacks the host cell machinery to establish a viral replication center, a process orchestrated by diverse viral proteins. The 5’ two-thirds of its genome (ORF1a and ORF1b) encode 16 non-structural proteins (nsps) that collectively drive viral replication, safeguard viral RNA, and antagonize host immune responses. For instance, nsp3 promotes the assembly of viral replication organelles (27–30) and cleaves key host factors such as IRF3 and ISG15, thereby dampening interferon expression and MDA5-mediated antiviral activity (31–33). Furthermore, the Mac1 domain of nsp3 exhibits mono-ADP-ribosylhydrolase activity, counteracting host-driven antiviral ADP-ribosylation (34). Equally critical is nsp13, an RNA helicase (35) that functions synergistically with the RNA-dependent RNA polymerase (RdRp) to facilitate proofreading via backtracking, thereby conferring viral resistance to nucleoside analogs (36–39). Beyond its helicase function, nsp13 possesses RNA 5’-phosphatase activity requisite for viral RNA capping, a modification that shields the genome from immune recognition (40–42). Independently, nsp13 further subverts host immunity by inhibiting the activation of STAT1/2, IRF3, and TBK1, effectively blocking interferon signaling pathways (43–47).

The 3’ one-third of the genome encodes structural and accessory proteins translated from subgenomic mRNAs. Structural proteins include the Spike (S), Nucleocapsid (N), Membrane (M), and Envelope (E) proteins. The S protein mediates entry and induces IRF3 degradation (38–53). The E and M proteins assist in S protein localization, form ion channels, and drive viral assembly (54–57). The M protein also antagonizes the type I interferon pathway by degrading TBK1 and restricting MAVS activation (45, 53, 58, 59). The N protein forms the viral nucleocapsid (54, 57, 60, 61) and disrupts host stress granules through phase separation (62, 63), while also inhibiting RIG-I, MAVS, and STAT nuclear translocation (64–68). The roles of accessory proteins (encoded by ORF3-ORF9) remain incompletely understood but are heavily implicated in immune evasion (69–73). **Figure 1** shows the translation of SARS-CoV-2 RNA after entering the host cell.

Viruses first recognize and enter specific host cells through particular mechanisms. Once inside, they exploit the host’s cellular machinery to replicate. Throughout this process, viral proteins facilitate replication while also interfering with the host’s immune response to prevent antiviral defense. Nonetheless, the precise functions of certain viral proteins remain to be further investigated.

## 4 Antiviral role of E3 ubiquitin ligases

With a basic framework of E3 ubiquitin ligases and the host-SARS-CoV-2 interaction landscape in place, both timely and imperative to investigate how host E3 ligases can be leveraged to counter viral infection. In the dynamic virus-host network, E3 ligases-central executors of ubiquitination-shape infection outcomes by selectively targeting host or viral proteins and altering their stability, activity, or subcellular localization. Dissecting the mechanisms by which specific E3 ligases modulate viral entry, replication, and antiviral immunity is therefore essential for understanding SARS-CoV-2 pathogenesis and for identifying actionable therapeutic targets.

In host defense against SARS-CoV-2, several E3 ligases act as positive regulators of innate immunity by sustaining antiviral signaling and directly restricting viral proteins. One representative example involves nsp16, a non-structural protein required for viral mRNA capping. The host E3 ligases UBR5 and MARCHF7 ubiquitinate nsp16 and promote its proteasomal degradation. Mechanistically, UBR5 predominantly catalyzes K48-linked ubiquitination, whereas MARCHF7 mediates K27-linked ubiquitination; both modifications suppress viral replication in cellular and mouse models. Notably, this activity extends to nsp16 variants from different viral strains, suggesting a potential breadth of antiviral coverage. These findings expand our understanding of how the ubiquitin-proteasome system contributes to antiviral defense and highlight nsp16-directed ubiquitination as a possible therapeutic entry point (74). Nsp5 (the main protease) is indispensable for polyprotein processing and viral replication, and it also promotes immune evasion by cleaving key host proteins involved in interferon signaling, inflammasome regulation, and stress-granule formation (e.g., RIG-I, MAVS, TAB1, NLRP12, and MAGED2). FBXO22 has been identified as a restriction factor that recognizes nsp5 and catalyzes K48-linked polyubiquitination at lysine residues 5 and 90, leading to proteasomal degradation of nsp5. Consistently, reducing FBXO22 expression decreases nsp5 ubiquitination, increases nsp5 stability, and enhances viral immune evasion. Clinical observations further indicate a negative correlation between FBXO22 expression and patient viral load, supporting a model in which FBXO22 limits infection by destabilizing a core viral enzyme (75). Nsp8, a cofactor for the RdRp complex, is targeted by TRIM22, an interferon-inducible E3 ligase. TRIM22 mediates the K48-linked ubiquitination and degradation of nsp8, effectively stalling viral RNA synthesis. However, the precise regulatory balance is delicate; excessive TRIM22 activity may trigger immunopathological damage, highlighting the safety risks associated with targeting E3 enzymes (76). The ORF6 protein is an important accessory protein of COVID-19, whose core function is to act as a potent inhibitor of the type I interferon signaling pathway. It can significantly block the synthesis of endogenous interferons in host cells and further inhibit the activation and expression of a series of downstream interferon-stimulated genes. The host protein PRPF19 binds to the CRL4B ubiquitin ligase complex (CUL4B/DDB1/RBX1) to specifically ubiquitinate and degrade ORF6. Relieving ORF6-mediated immunosuppression restores interferon signaling and reduces lung pathology in animal models, highlighting CUL4B activation as a viable antiviral strategy (77). Similarly, the ORF9b protein, another interferon antagonist, is targeted for K48-linked degradation by three independent host E3 ligases: HUWE1, UBR4, and UBR5. This degradation limits the virus’s ability to suppress host immunity (78). Structural proteins can also be subject to ubiquitin-mediated restriction. The E protein is multifunctional in viral assembly and budding and can influence host immunity (including inflammasome activation), making it an attractive target for antiviral development. RNF5 has been reported to ubiquitinate and promote degradation of the E protein, thereby suppressing viral replication; RNF5 expression has also been associated with patient age and disease severity, raising the possibility of prognostic utility, and the reported antiviral effect appears to extend across multiple coronavirus variants (2). In a separate line of evidence, the E protein can be regulated through host control of the ubiquitin-proteasome pathway, and RNF185 was identified as a key E3 ligase that colocalizes with the E protein in the endoplasmic reticulum and promotes its ubiquitination-dependent degradation. Consistently, RNF185 knockdown increases SARS-CoV-2 titers in cell models, suggesting that strengthening RNF185-E protein restriction may offer another antiviral avenue (79). Furthermore, host transcription factor EGR1 upregulates interferon-regulated antiviral protein (IRAV), activating the MARCH8-mediated lysosomal degradation of the viral N protein (80). Intriguingly, environmental factors can also manipulate E3 ligases; for example, benzo[a]pyrene in tobacco promotes Skp2-mediated degradation of the ACE2 receptor, offering a potential molecular explanation for the altered COVID-19 susceptibility observed in some smokers (81).

**Figure 2:**
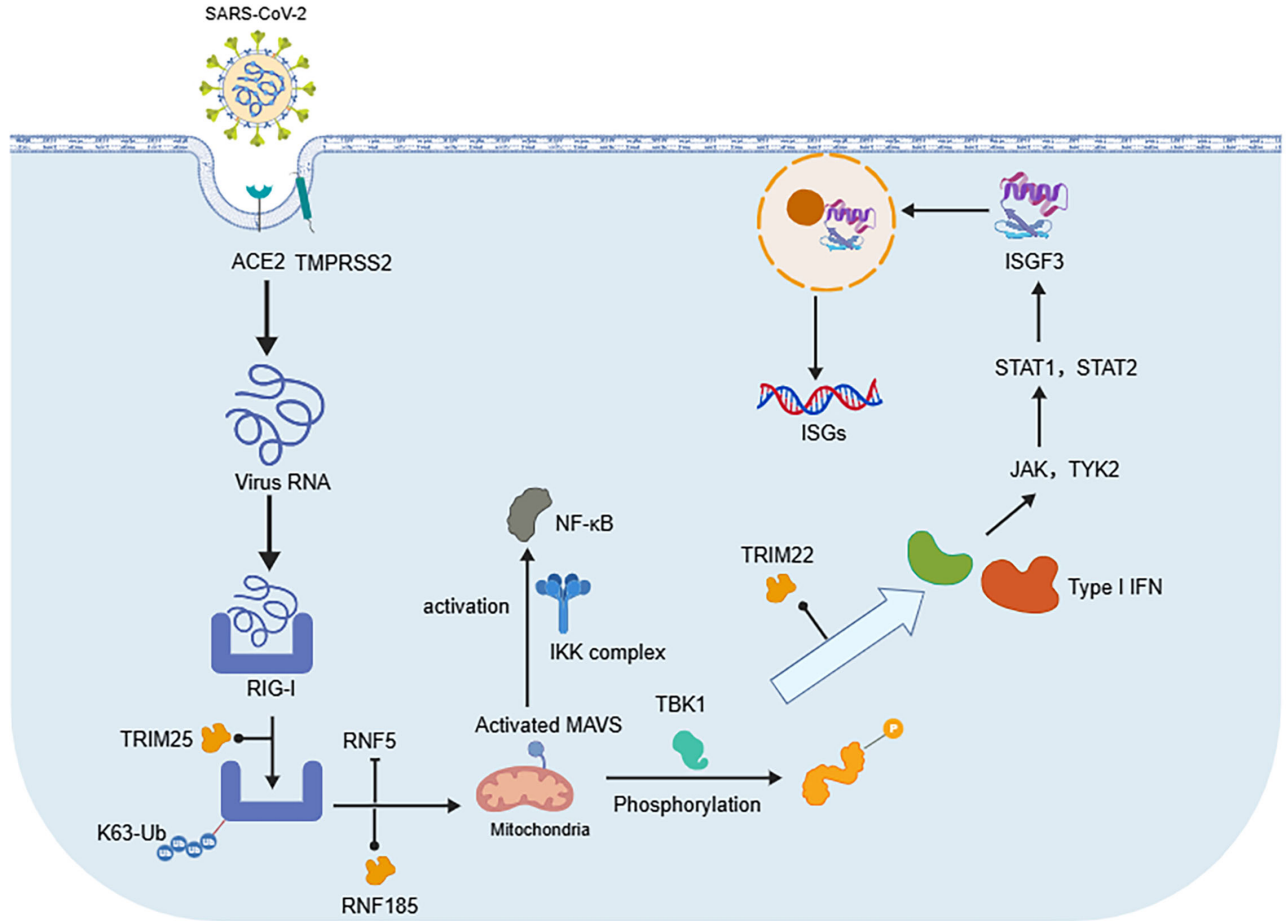
Upon viral entry into the host cell, the viral genetic material is recognized by RIG-I, which then activates MAVS following ubiquitination by TRIM25. MAVS in turn activates the IRF3 and NF-κB pathways. Activation of these pathways promotes the transcription of antiviral genes, leading to the production of large amounts of type I interferons (IFNs). Once secreted, IFNs activate JAK1 and TYK2, which subsequently activate STAT1 and STAT2 to form the ISGF3 complex. This complex translocates into the nucleus and induces the expression of ISGs, thereby establishing an antiviral state in the cell (3, 82–84). This image was created with BioGDP.com.

The coronavirus life cycle is highly dependent on the host, prompting the virus to converge on the same host nodes. First is the TRIM25-RIG-I axis: TRIM25 is responsible for the K63-linked ubiquitination of RIG-I to activate the interferon system. The N protein of SARS-CoV, ORF6 and N protein of SARS-CoV-2, as well as accessory proteins of MERS, can all target this pathway (13). Meanwhile, the MARCH and RNF families are broadly shared; they are core modules of the host defense against RNA viruses. Therefore, coronaviruses continuously undergo convergent evolution to target them. The role of E3 ubiquitin ligases in the innate immune signaling pathway is illustrated in **Figure 2**.

## 5 Proviral role of E3 ubiquitin ligases

Not all host E3 ubiquitin ligases are beneficial to antiviral defense. During SARS-CoV-2 infection, the virus can exhibit highly selective “molecular hijacking” of the host ubiquitination machinery. E3 ligases normally function in protein quality control by tagging specific substrates for degradation or functional rewiring; however, viral proteins can recruit or redirect particular E3 ligases to ubiquitinate key antiviral factors, thereby weakening innate immune defenses and creating a cellular environment permissive for replication.

A representative example is RNF5. As discussed above, RNF5 can restrict SARS-CoV-2 by ubiquitinating the E protein and promoting its degradation. In contrast, RNF5 has also been reported to ubiquitinate the M protein at lysine 15 (K15), which enhances M-E interaction. Because coordinated M-E interactions facilitate efficient virion assembly and contribute to the production of uniformly sized viral particles, this RNF5-dependent modification may ultimately support viral maturation (85). Beyond RNF5, multiple additional E3 ligases have been implicated as proviral regulators—including HUWE1, NEDD4, PIAS4, STUB1, TRIM6, TRIM22, UBE4B, USP5, WWP1, and ZYG11B—through mechanisms such as promoting viral replication, stabilizing ACE2, suppressing interferon signaling, or perturbing cellular architecture (86). A summary of E3 ubiquitin ligases and their targeted substrates in the context of SARS-CoV-2 infection is provided in **Table 1**.

**Table 1:** Correspondence between E3 ubiquitin ligase and targeted molecules.

E3 ubiquitin ligase	Molecule targeted by E3 ubiquitin ligase	The role of the targeted molecule	Effect on viral infections
MARCH8 (1), RNF5 (2) RNF185 (79)	Envelope protein	mediate virus assembly and release, and construct replicating membranes	Inhibition
UBR5, MAECHF7 (74)	nsp16	Mediated viral mRNA capping	Inhibition
CRL4B (77)	ORF6	An important accessory protein that acts as a powerful inhibitor of the type I interferon signaling pathway	Inhibition
HUWE1, UBR4, UBR5 (78)	ORF9b	Inhibits the activation of type I and III interferon signaling pathways, which have antiviral effects in host cells	Inhibition
FBXO22 (75)	nsp5	Responsible for cleaving viral polymers and is essential for viral replication	Inhibition
TRIM22 (76)	nsp8	A key cofactor of RdRp, which is involved in the formation of the core replication-transcription complex (RTC), is essential for the synthesis, recognition, and replication of viral RNA.	Inhibition
Skp2 (81)	ACE2	The main cellular receptor for SARS-CoV-2	Inhibition
RNF5 (85)	Membrane proteins	It mediates the plasticity of virus assembly and participates in immune regulation	promotion

Host cells can be broadly divided into two types of E3 ubiquitin ligases: conserved E3s and adaptive E3s. Adaptive E3s evolve rapidly, possess strong antiviral functions, and engage in an arms race with viruses. They are often induced by IFN, undergo rapid evolution, exhibit clear positive selection, and show considerable species-specific differences. Most of them belong to the TRIM family. In essence, these E3s are weapons specifically evolved by the host to combat viruses. In contrast, other E3s that cannot easily mutate and are under strong purifying selection have more fundamental functions and are readily co-opted by coronaviruses. These are typically important cellular functional proteins involved in processes such as membrane transport and autophagy. Overall, SARS-CoV-2 and MERS-CoV tend to potently antagonize IFN-related E3s, whereas endemic coronaviruses maintain milder, long-term adaptive interactions (87–89).

## 6 Other ubiquitin-like modifications of SARS-CoV-2

Beyond canonical ubiquitin-mediated degradation, the host machinery also employs ubiquitin-like (UBL) modifications-most notably SUMOylation and ISGylation-to regulate viral pathogenesis. SARS-CoV-2 has evolved mechanisms to both exploit these modifications for its own benefit and actively subvert them to escape immune surveillance.

First, SUMOylation has emerged as a critical regulator of viral assembly and localization. The SARS-CoV-2 Nucleocapsid (N) protein is a primary target for SUMOylation at residues K61, K65, and K347. Functionally, these modifications promote N protein oligomerization, thereby facilitating viral RNA packaging and virion assembly. Notably, SUMOylation at K65 is indispensable for the nuclear translocation of the N protein, underscoring the role of this PTM in governing viral subcellular localization. Given its pivotal contribution to the viral life cycle, the host SUMOylation machinery represents a promising target for therapeutic intervention (90).

In parallel, the host innate immune response leverages ISGylation to restrict viral infection. Upon activation of the interferon signaling pathway, the highly induced UBL protein ISG15 is covalently conjugated to target proteins via an enzymatic cascade. The host E3 ligase HERC5 specifically ISGylates the SARS-CoV-2 N protein at K261. This modification critically inhibits N protein dimerization and oligomerization, disrupts RNA synthesis, and sensitizes the virus to interferon treatment (77). However, SARS-CoV-2 counteracts this defense via its papain-like protease (PLpro), which functions as a potent de-ISGylase and deubiquitinase. PLpro preferentially cleaves K48-linked ubiquitin chains and ISG15 conjugates through a bivalent binding mechanism, effectively dampening innate immunity. Mutations in key PLpro residues compromise this activity, further highlighting the structural basis of SARS-CoV-2 immune evasion strategies (92–94).

In infections with coronaviruses such as SARS-CoV and SARS-CoV-2, ubiquitination, SUMOylation, and ISGylation all belong to the ‘small protein modifier (UBL) modification’ system. Structurally, each depends on an E1-E2-E3 enzymatic cascade and covalently links to substrate proteins via lysine residues. Functionally, all three are deeply involved in innate immune regulation, albeit with different emphases: ubiquitination primarily regulates the activation and degradation of signaling proteins such as RIG-I, MAVS, and TBK1; SUMOylation is more involved in nuclear transcriptional regulation, stress granule formation, and stabilization of the viral replication complex; and ISGylation is an important IFN-induced antiviral modification that enhances MDA5/RIG-I activity and restricts viral replication. The SARS-CoV-2 PLpro possesses both deubiquitinase and deISGylase activities, enabling it to remove K63-linked ubiquitin and ISG15, thereby suppressing IFN production. This indicates that the virus systemically remodels the UBL network to achieve immune evasion. Therefore, although these three modifications share similar structural mechanisms, they perform three core functions during coronavirus infection: ‘signal amplification,’ ‘transcriptional/stress regulation,’ and ‘IFN-mediated antiviral restriction’ (95–97).

## 7 Discussion

This review systematically summarizes the pivotal role of E3 ubiquitin ligases in SARS-CoV-2 infection, positioning them as central hubs within the host-virus interaction network. E3 ligases exhibit dichotomous functions: on one hand, they target viral proteins-such as nsp16, ORF6, and nsp5 (74, 75, 77) for ubiquitination and degradation, thereby restricting viral replication and bolstering interferon signaling. On the other hand, certain E3 ligases are hijacked by the virus to degrade host antiviral factors or stabilize viral protein interactions, facilitating immune evasion and viral maturation. Furthermore, ubiquitin-like modifications, including SUMOylation and ISGylation (90–94) have emerged as critical regulatory layers that govern viral protein stability and host immune responses. Collectively, these findings provide essential insights into viral pathogenesis and highlight the therapeutic potential of targeting E3 ligases and ubiquitin-like modification pathways.

While current studies have elucidated the mechanisms of several E3 ligases, the regulatory balance between “antiviral” and “proviral” E3s, as well as the specific targets and logic governing ubiquitin-like modifications, remain to be fully explored. Significant knowledge gaps persist regarding the complex interplay between viral proteins and host factors. Future research must leverage precise molecular tools to define the substrate specificity and regulatory circuits of key enzymes, and to decipher the synergistic or antagonistic relationships between distinct modification pathways.

Furthermore, the clinical translation of E3 ligases as therapeutic targets faces three major bottlenecks. First, specificity: most E3 ligases possess broad substrate spectrums, and their targeted modulation may disrupt normal host physiology (e.g., targeting Skp2 could interfere with cell cycle regulation). Second, viral escape: This calls for a deeper understanding of how SARS-CoV-2 variants reshape their interaction networks with host E3 ligases and ubiquitin-like modifications. Key considerations include how mutations alter lysine exposure or degron structures on viral proteins, thereby affecting host E3 recognition and ubiquitination of viral proteins, as well as how they reshape innate immune regulation to provide the virus with a longer replication window. Mutations in viral ubiquitination sites may abolish E3 recognition, compromising the broad-spectrum efficacy of therapeutic agents. Third, model discrepancy: existing studies rely heavily on cell lines or young animal models, whereas severe COVID-19 predominantly affects the elderly and those with comorbidities. The expression profiles, activities, and regulatory networks of E3 ligases in these patients may differ significantly from experimental models, potentially leading to translational failure.

In summary, the antiviral functions of E3 ubiquitin ligases offer a novel perspective for COVID-19 therapy. However, the simplistic paradigm that “identifying an E3 ligase that degrades a viral protein equates to finding a therapeutic target” must be critically re-evaluated. Future research should prioritize (1): elucidating the molecular switches that govern the functional transition of E3 ligases between antiviral and proviral roles (2); defining the mechanisms by which viruses evade E3-mediated degradation (3); assessing the tissue specificity and safety profiles of E3-targeted interventions; and (4) validating the correlation between E3 ligase expression and disease prognosis in clinical cohorts. Only through a comprehensive understanding of the complexity, duality, and evolutionary dynamics of E3 ligase networks can clinically viable antiviral strategies be developed.

## 8 Methods

We searched for literature on PubMed using keywords such as “E3 ubiquitin ligases” and “SARS-CoV-2”, primarily referring to articles published in the past six years to cover as comprehensively as possible the research related to SARS-CoV-2 and E3 ubiquitin ligases. In addition, we manually screened the reference lists of the included studies to supplement important literatures that might have been missed by database retrieval.
